# Knowledge, Attitudes, and Practices of Dentists Regarding the Diagnosis and Management of White Spot Lesions

**DOI:** 10.3390/dj14060365

**Published:** 2026-06-12

**Authors:** Nina Novozhilova, Anastasia Mun, Maria Polyakova, Irina Makeeva, Anna Mikheikina, Inna Sokhova, Alexandr Zaytsev, Ksenia Babina

**Affiliations:** 1 Department of Therapeutic Dentistry, Sechenov First Moscow State Medical University (Sechenov University), 119991 Moscow, Russia; mun_a_s@student.sechenov.ru (A.M.); makeeva_i_m@staff.sechenov.ru (I.M.); sokhova_i_a@staff.sechenov.ru (I.S.); babina_k_s@staff.sechenov.ru (K.B.); 2 Institute of Foreign Languages for Professional Purposes, Sechenov First Moscow State Medical University (Sechenov University), 119991 Moscow, Russia

**Keywords:** dental caries, tooth demineralization, dentists, health knowledge, attitudes, practice, tooth remineralization, resin infiltration, microabrasion

## Abstract

**Background**: The management of white spot caries lesions (WSCLs) presents a clinical challenge due to a lack of standardized protocols. This study aimed to evaluate the knowledge, attitudes, and practices of dental practitioners in Russia regarding the diagnosis and treatment of WSCLs. **Methods**: A cross-sectional online survey was conducted from October 2024 to July 2025 among 397 dental professionals in Russia. A validated questionnaire assessed four domains: demographics, knowledge, attitudes, and clinical practices concerning WSCL management. Data were analyzed using descriptive statistics, Kruskal–Wallis tests, and Spearman’s correlation. **Results**: The majority of respondents were females (83%), with over half having less than five years of experience (55%). A majority of the practitioners (62.5%) demonstrated a good level of knowledge (mean score 12.8 ± 2.2). Knowledge was significantly better among female practitioners (*p* < 0.001) and inversely correlated with years of clinical experience (*p* < 0.001). Attitudes towards minimally invasive treatment were mainly positive (mean score 13.5 ± 2.6), with 86% of respondents showing a positive score (>10 points). While awareness of minimally invasive techniques like resin infiltration (84%) and remineralization (79.1%) was high, clinical practice varied. Remineralization was the most frequently used treatment (68% used casein phosphopeptide–amorphous calcium phosphate and 62% used fluoride), whereas resin infiltration was used by 46% and microabrasion by only 5% of the respondents. A majority of dentists (52%) preferred monitoring lesions rather than immediate intervention. **Conclusions**: Russian dental practitioners possess good theoretical knowledge and positive attitudes toward contemporary, minimally invasive management of WSCLs. However, a significant gap exists between knowledge and clinical practice, particularly concerning the use of resin infiltration and microabrasion. These findings underscore the need for continuous education on the topic.

## 1. Introduction

White spot caries lesions (WSCLs) represent incipient enamel caries. According to the International Caries Detection and Assessment System (ICDAS II), these lesions are classified as codes 1 and 2 characterized by substantial mineral loss within subsurface enamel while maintaining a relatively intact surface layer [1]. Clinically, the enamel surface appears smooth and hard upon tactile probing [2,3]. However, failure to provide timely intervention leads to irreversible structural degradation and the formation of a carious cavity, requiring invasive treatment (preparation and restoration) [1].

One significant factor contributing to a high prevalence rate of WSCLs is the widespread use of fixed orthodontic appliances [4,5]. Orthodontic hardware creates additional areas that facilitate the accumulation and maturation of cariogenic biofilm. This results in localized pH drops below the critical threshold (5.5), triggering the demineralization process [6,7] that may lead to cavity formation. Beyond the risk of caries progression, focal demineralization is one of the causes of intrinsic tooth discoloration [8], frequently becoming the primary source of patient dissatisfaction following orthodontic correction.

Currently, no universal gold standard exists for WSCL management, with preference given to minimally invasive concepts, including remineralization, microabrasion, and resin infiltration. Remineralization therapy aims to restore the enamel crystal lattice through the application of fluorides [9,10], casein phosphopeptide–amorphous calcium phosphate (CPP–ACP) complexes [11,12,13,14], or synthetic hydroxyapatite [15,16,17]. Remineralization of WSCLs promotes the restoration of enamel mineral structure [18,19] and improves its optical properties [20,21] by reducing subsurface porosity, which decreases light scattering and brings the appearance of the affected area closer to that of sound enamel.

Alternatively, microabrasion may be used to manage superficial demineralization and discolorations by mechanical removal of the affected layers with hydrochloric acid and abrasive paste [22,23,24]. A third modality is resin infiltration using low-viscosity resins. Resin infiltration is a micro-invasive technique in which a light-cured resin is applied to the porous enamel of a WSCL after etching with hydrochloric acid and drying with ethanol [25]. The resin penetrates the body of the lesion, occluding the microporosities and thereby masking the opacity of the lesion by approximating the refractive index of the infiltrated area to that of sound enamel [26].

Despite the scientific evidence supporting the use of the aforementioned approaches, dental professionals are often reluctant to treat incipient carious lesions. Moreover, the choice of a management strategy frequently remains subjective. This results in a delay between diagnosis and active treatment. Therefore, the aim of this study was to evaluate the knowledge of dental practitioners regarding the diagnosis and treatment of WSCLs and to analyze their attitudes toward contemporary non-invasive approaches and their clinical use.

## 2. Materials and Methods

This study received formal ethical approval from the Ethics Committee of Sechenov University, Moscow, Russia (Protocol No. 24-24). The cross-sectional online survey was carried out between October 2024 and July 2025. Participation was entirely voluntary; by completing and submitting the survey, respondents provided their informed consent for both study involvement and the subsequent publication of anonymized, aggregated findings.

A questionnaire was developed in Russian based on the contemporary literature on the topic [27,28,29,30].

The instrument’s content validity was established through expert review (*n* = 3), followed by a pilot study among 30 randomly selected dental professionals who were excluded from the final sample. The survey for piloting was distributed via professional social media platforms (Telegram, VK). Each item was graded as “clear” (unambiguous, easy to understand, having a single obvious meaning, and with no answer options missing) or “unclear” (confusing, vague, ambiguous, open to multiple interpretations, or with some answer options missing). After the clarity and completion time were assessed, the questionnaire was refined based on pilot feedback to ensure optimal comprehension. Two items were revised and three answer options were introduced. A second round of piloting revealed no unclear items.

The final structured 3 min survey comprised four domains: demographics, knowledge, attitudes, and clinical practices regarding WSCL treatments. The demographic section included data on age, gender, professional experience, workplace, and specialization.

The knowledge domain comprised six (three single-choice and three multiple-choice) items evaluating diagnosis and treatment protocols for WSCLs. Correct responses were assigned one point, while incorrect answers received zero. For the multiple-choice items with multiple correct options, each correct answer earned one point; thus, the total score for each item depended on the number of the correct answers selected. The maximum aggregate score for the knowledge section was 17. Knowledge levels were stratified based on percentage thresholds: poor (≤50%, ≤8 points), fair (50–75%, 9–12 points), and good (>75%, >12 points).

The attitude domain evaluated respondents’ perspectives on WSCL management using a five-point Likert scale. This section comprised one positively phrased item (ranging from 5—“strongly agree” to 1—“strongly disagree”) and three reverse-scored negative statements (1 ranging from 1—“strongly agree” to 5—“strongly disagree”). Cumulative scores were defined as “positive” overall attitude for scores exceeding 50% (>10 points) or “negative” overall attitude for scores at or below the 50% threshold (≤10 points).

The practice domain comprised four items assessing WSCL diagnosis and management. There were two single-choice and two multiple-choice questions.

Data collection was accomplished through Google Forms. The link to the questionnaire was disseminated through professional social media platforms (Telegram, VK). All data were collected anonymously and treated confidentially.

The targeted samples included practitioners working in different fields of dentistry in the Russian Federation. Based on the data from the Federal State Statistics Service, the total professional population was estimated at approximately 72,000 individuals [31].

A minimum sample size of 385 respondents was determined, assuming a 95% confidence interval and a 5% margin of error, according to the following formula:

$$\mathrm{Sample}\;\mathrm{size}\;=\;\frac{z^{2}\times p\left(1-p\right)}{e^{2}},$$

where:

*z* (z-score) = 1.96 (95% CI);

*p* (anticipated proportion) = 0.5;

*e* (margin of error) = 0.05.

Data management was conducted using Microsoft Excel (version 16.71) and R (version 4.2.3) within the RStudio environment (version 2023.03.0). The following R packages were used: “doBy,” “rstatix,” and “stats.” Descriptive statistics were reported as means and standard deviations and medians with interquartile ranges (25th–75th percentiles) for continuous variables, while categorical data were expressed as counts and percentages. The Shapiro–Wilk test was used to evaluate the normality of quantitative distributions (knowledge, attitude, and practice scores). Given the non-normal distribution of the data, group comparisons were performed using the Kruskal–Wallis rank-sum test, followed by Dunn’s post hoc test for pairwise comparisons. Proportional differences were assessed via Fisher’s exact test. Spearman’s rank correlation coefficients were calculated to examine the associations between knowledge, attitude, and practice scores.

## 3. Results

A total of 397 dental professionals participated in the study. The gender distribution was predominantly female, accounting for 82.6% (*n* = 328) of the sample (Table 1). Regarding professional specialization, the largest groups were dental therapists (35.5%, *n* = 141) and general dentists (32.2%, *n* = 128), followed by pediatric dentists (12.3%, *n* = 49). More than half of the respondents (54.9%, *n* = 218) were early-career professionals with less than five years of experience. In contrast, experienced clinicians with over 20 years of practice comprised 14.1% (*n* = 56) of the participants. Most respondents (75.8%, *n* = 301) were employed in private dental clinics, while 24.2% (*n* = 96) worked in government-funded clinics. 

The assessment of participants’ theoretical knowledge regarding WSCLs revealed an overall high level of competence (Table 2). The mean knowledge score for the survey population was 12.8 ± 2.2 out of a maximum of 17 points, with a median score of 13 (IQR 11–14). The majority of the respondents (62.5%, *n* = 248) demonstrated a “Good” level of knowledge, while only a small proportion (2.8%, *n* = 11) was categorized as having “Poor” knowledge.

Female practitioners achieved significantly higher scores (13.1 ± 2.0) compared to male practitioners (11.2 ± 2.2), (*p* = 2.897 × 10^−10^). Knowledge levels also differed significantly across the specialties (*p* < 0.001). The highest mean scores were achieved by pediatric dentists and dental surgeons (13.7 ± 1.9 and 13.5 ± 0.5, respectively), whereas prosthodontists scored the lowest (10.8 ± 0.4). Interestingly, we found a significant inverse relationship between years of practice and knowledge scores (*p* < 0.001). Respondents with less than 5 years of experience showed the highest mean scores (13.1 ± 2.0), while those with over 20 years of experience showed the lowest mean scores (11.5 ± 3.0).

Half of the respondents (50.4%) correctly identified WSCLs as being characterized by subsurface demineralization (Table 3). Visual examination (85.6%) and staining (82.6%) were the most recognized diagnostic methods, while some participants believed that polarization microscopy could be used in a clinical setting (30.5%). The respondents demonstrated a high awareness of minimally invasive options, specifically infiltration (83.9%) and remineralization (79.1%). A vast majority (91.4%) believed that the formation of a carious cavity is the main complication in the absence of treatment. Also, 74.1% of the surveyed dentists correctly identified microabrasion as the use of an abrasive and hydrochloric acid mixture, and 69.5% of them accurately identified the infiltration technique as etching followed by filling of micropores with resin.

The analysis of participants’ responses to specific items in the attitudes section (Table 4) revealed a strong inclination towards minimally invasive dentistry, though some divergence in opinions was also noted. More than half of the practitioners (53.1%) indicated that WSCLs always require active treatment, whereas 38.8% favored a more observational or case-specific approach. A substantial majority of the respondents (73.5%) disagreed or strongly disagreed with the statement that remineralization is ineffective. The majority (54.1%) disagreed that the infiltration technique significantly increases the probability of pigmented plaque formation, while 31.5% of participants remained neutral on this issue. Opinions were divided regarding the aggressiveness of microabrasion: 43% of the respondents viewed the procedure as overly aggressive, while 31.8% considered it a safe and appropriate modality for WSCL management.

The surveyed dentists generally demonstrated positive attitudes toward the active management of WSCLs, with a total mean score of 13.5 ± 2.6 and a median of 14 (IQR 12–15) (Table 5). Overall, 86.4% (*n* = 343) of the respondents exhibited a “Positive” attitude toward evidence-based preventive and minimally invasive protocols. There were significant differences in attitude scores among specialties (*p* < 0.001). Dental surgeons (14.8 ±0.9) and pediatric dentists (14.4 ± 2.6) had the highest mean scores, while prosthodontists (11.5 ± 1.2) had the lowest mean scores. Clinical experience was found to be significantly associated with attitudes toward WSCLs management (*p* < 0.001). Dental practitioners with 5–10 years of experience demonstrated the highest mean score (14.0 ± 2.4), while those with over 20 years of clinical practice showed lower attitude scores (12.2 ± 2.0).

The survey evaluated the self-reported clinical behaviors of practitioners regarding the diagnosis and treatment of WSCLs (Table 6). While the majority of our respondents frequently encountered this pathology, their diagnostic and therapeutic preferences varied. Almost a quarter (24.2%) diagnosed these lesions in less than 10% of cases and 16.1% identified them in more than 40% of their patients, while only 1.8% reported never diagnosing WSCLs. Visual examination was the most prevalent diagnostic method, utilized by 89.9% of the practitioners, followed by probing (59.4%) and staining with methylene blue (57.2%). Interestingly, 1.8% of the respondents reported using polarization microscopy, which cannot be applied to vital teeth.

Remineralization protocols were the most common treatment choice, with 67.5% of dentists using CPP-ACP, 62.2% using fluoride-based agents, and 26.4% using HAP formulations. Resin infiltration (ICON, DMG, Hamburg, Germany) was practiced by 46.1% of the respondents, while microabrasion was used by only 5.0%. Moreover, 51.9% preferred monitoring the lesion rather than immediate intervention. When treating lesions in esthetically important zones, resin infiltration (35.0%) and composite restoration (15.4%) were the most preferred approaches. At the same time, the use of remineralizing agents (CPP-ACP and fluoride) decreased to 14.6% and 10.3%, respectively, when esthetic outcome was prioritized.

## 4. Discussion

Despite a high prevalence of WSCLs, there is still no universal approach regarding the management of these lesions. Remineralization, microabrasion, resin infiltration, and combinations of these methods are available as possible treatment options [32]. The aim of our study was to assess the knowledge, attitude, and practices of dental professionals in Russia as regards the management of WSCLs. The survey revealed a significant difference between the practitioners’ theoretical knowledge and their clinical practice. Although most respondents demonstrated good knowledge and a positive attitude, their practical application of modern treatment modalities was limited. This result is in agreement with a systematic review by de Moura et al., who found that while dentists’ knowledge of minimal intervention dentistry was acceptable, their practice required improvement [33].

In our survey, dental practitioners showed a good knowledge of WSCL management, with a mean score of 12.8 out of 17. This level of knowledge is higher compared to that reported in other studies [27,34,35]. A study conducted among dental practitioners in India found that while all professionals were aware of WSCLs, their specific knowledge of diagnostic approaches was low [34]. A survey of Pakistani dentists described the awareness regarding WSCL management as low [27], while a Saudi study reported a generally low level of knowledge regarding resin infiltration [35].

A majority of the respondents correctly identified minimally invasive treatment options such as resin infiltration (83.9%) and remineralization (79.1%). However, only half of the respondents (50.4%) correctly identified subsurface demineralization as the defining characteristic of a WSCL. This may indicate a gap in the fundamental understanding of the lesion pathology. Furthermore, 30.5% of the clinicians believed that polarization microscopy, a laboratory technique, could be used for clinical diagnosis, which shows some confusion between clinical and research methods.

We found a significant negative correlation between the years of clinical experience and knowledge scores (*p* < 0.001): dental practitioners with less than 5 years of experience demonstrated the highest scores. This finding is in agreement with the findings of a systematic review by Choudhry et al., who reported a similar relationship between physicians’ experience and quality of care [36]. This may be due to the fact that recently trained professionals are more familiar with current evidence-based protocols. Similarly, a study by Weyland et al. found that younger orthodontists were more concerned with WSCL prevention than their senior colleagues [30]. Another significant finding was that female practitioners had higher knowledge scores compared to male practitioners (*p* < 0.001). This gender difference in health-related knowledge has also been reported in a study by Murphy et al. [22]. However, these findings should be interpreted with caution due to the gender imbalance in the survey population.

In our survey, more than 80% of the dental practitioners demonstrated a positive attitude toward minimally invasive management. No significant differences were found between most of the study subgroups in this parameter. More than half of the respondents (53%) agreed that WSCLs always require active treatment. However, a large proportion (39%) preferred a more observational approach. This is in agreement with the practice data, where 52% of clinicians reported a preference for monitoring lesions rather than immediate intervention. This conservative preference is consistent with the principles of minimally invasive dentistry but may also show a lack of confidence in performing more definitive treatments.

A substantial difference between knowledge and practice was observed for resin infiltration. Although awareness of this method was high (84%), its use in practice was reported by only 46%. The use of resin infiltration by the participants of our study turned out to be higher than that reported among orthodontists in Germany (21%) [30] and general practitioners in South India (16%) [34]. At the same time, microabrasion was the least popular technique, used by only 5.0% of the respondents. This low use may be related to a negative attitude, as 43% of the practitioners believed that the procedure was “too aggressive.” This perception is a significant barrier, despite evidence supporting the use of microabrasion for masking WSCLs [22].

Overall, the knowledge–practice gap may be attributed to several reasons. On the one hand, senior practitioners may be reluctant to implement the latest research (infiltration, microabrasion, etc.) into routine practice, relying instead on outdated approaches (e.g., fluoride varnish). On the other hand, recent graduates, despite their good theoretical background, often experience difficulties when transferring it into practical, real-world scenarios. Moreover, advanced dental materials for WSCL treatment are relatively expensive, which makes their use uneconomical without a high patient turnover. Failure to use non-invasive methods for WSCL management may result in adverse clinical outcomes. Without timely and accurate treatment, WSCLs may progress to further demineralization and the formation of a carious cavity, requiring invasive intervention (“drilling and filling”). Therefore, it is essential to incorporate more practical, preclinical training and focus on independent decision-making during undergraduate studies. Moreover, it is necessary to provide consistent opportunities for senior dentists to update their skills on new techniques.

We readily acknowledge several limitations to our survey. First, it was a cross-sectional study which assessed a single timepoint and, thus, cannot establish causality. Second, the use of self-reporting data could lead to response bias and affect the accuracy of the findings. Third, convenience sampling was used, covering only individuals who use the internet and social platforms, while excluding the offline population; this resulted in an imbalanced survey population (with a predominance of early-career specialists). Furthermore, women usually demonstrate greater interest and willingness to participate in surveys, which likely contributed to a higher proportion of female respondents in the study sample. This may limit the generalization of the findings to the entire population of dentists in Russia.

In conclusion, dental practitioners in Russia showed good knowledge of WSCL management and a positive attitude toward modern, minimally invasive approaches. However, there is a gap between knowledge and clinical practice. The low use of techniques such as resin infiltration and microabrasion, together with the inverse correlation between experience and knowledge, shows a need for continuing education programs on the topic.

## Figures and Tables

**Table 1:** Sociodemographic and professional characteristics of the study population.

Sociodemographic Data	Count, *n*	Percentage, %
Gender		
Male	69	17.4
Female	328	82.6
Specialty		
Dental therapist	141	35.5
Pediatric dentist	49	12.3
Orthodontist	29	7.3
General dentist	128	32.2
Dental surgeon	11	2.8
Prosthodontist	11	2.8
Dental hygienist	28	7.1
Years of clinical experience		
<5	218	54.9
5–10	85	21.4
11–20	38	9.6
>20	56	14.1
Clinic		
Private	301	75.8
Government-funded	96	24.2

**Table 2:** Knowledge scores regarding WSCL management.

Sociodemographic Data	Knowledge (Max 17)	
	Mean (sd)	Median (IQR)
Total	12.8 (2.2)	13 (11; 14)
Gender		
Male	11.2 (2.2)	11 (9; 13)
Female	13.1 (2.0)	14 (12; 14)
	Kruskal–Wallis chi-squared = 39.743, df = 1, *p*-value = 2.897 × 10^−10^	
Specialty		
Dental therapist	12.8 (2.2) ^a^	13 (11; 14)
Pediatric dentist	13.7 (1.9) ^b^	14 (13; 15)
Orthodontist	13.2 (1.8) ^a^	14 (13; 14)
General dentist	12.4 (2.5) ^a^	13 (12; 14)
Prosthodontist	10.8 (0.4) ^c^	11 (11; 11)
Dental surgeon	13.5 (0.5) ^a,b^	13 (13; 14)
Dental hygienist	13.1 (2.0) ^a^	14 (12; 14)
	Kruskal–Wallis chi-squared = 27.341, df = 6, *p*-value < 0.001	
Years of clinical experience		
<5	13.1 (2.0) ^a^	14 (12; 14)
5–10	12.8 (2.1) ^a^	13 (11; 14)
11–20	12.9 (1.5) ^b^	13 (11.25; 14)
>20	11.5 (3.0) ^a,b^	12 (10; 13)
	Kruskal–Wallis chi-squared = 16.302, df = 3, *p*-value < 0.001	
Level, *n* (%)		
Poor	11 (2.8)	
Fair	138 (34.7)	
Good	248 (62.5)	

IQR—interquartile range; sd—standard deviation; df—degrees of freedom, ^a,b,c^—mean values within the same column followed by different superscript letters differ significantly; means sharing a common letter are not significantly different.

**Table 3:** Frequency distribution of responses to specific knowledge items concerning WSCL characteristics, diagnosis, and treatment.

Item	Variants	Answers, *n* (%)
WSCLs are characterized by	Subsurface demineralization	200 (50.4)
	Surface demineralization	194 (48.9)
	Disturbances of enamel formation	3 (0.7)
	Enamel necrosis	—
Minimally invasive treatment for WSCLs includes	Infiltration	333 (83.9)
	Microabrasion	220 (55.4)
	Remineralization	314 (79.1)
	Whitening	29 (7.3)
	Composite restoration	24 (6.0)
Diagnostic methods for WSCLs include	Staining	328 (82.6)
	Fluorescent diagnosis	255 (64.2)
	Polarization microscopy	121 (30.5)
	Visual examination	340 (85.6)
	Probing	241 (60.7)
Possible outcomes of WSCLs in the absence of treatment	Stabilization of the process	295 (74.3)
	Full remineralization	148 (37.3)
	Formation of a carious cavity	363 (91.4)
	Formation of enamel erosion	40 (10.1)
Microabrasion includes	Etching the surface of the lesion, then filling the micropores with resin	28 (7.0)
	Treatment of the lesion surface with a mixture of abrasive and hydrochloric acid	294 (74.1)
	Filling enamel micropores with minerals	25 (6.3)
	Treatment with hydrogen peroxide or carbamide	17 (4.3)
	I don’t know	33 (8.3)
Infiltration includes	Etching the surface of the lesion, then filling the micropores with resin	276 (69.5)
	Treatment of the lesion surface with a mixture of abrasive and hydrochloric acid	6 (1.5)
	Filling enamel micropores with minerals	89 (22.4)
	Treatment with hydrogen peroxide or carbamide	3 (0.8)
	I don’t know	23 (5.8)

WSCLs—white spot caries lesions.

**Table 4:** Distribution of participants’ responses to attitude statements on WSCL management.

Item	Respondents’ Answers *n* (%)				
	SA	A	N	D	SD
WSCLs always require treatment *	82 (20.6)	129 (32.5)	32 (8.1)	119 (30.0)	35 (8.8)
Remineralization is ineffective for WSCL treatment **	21 (5.3)	45 (11.4)	39 (9.8)	176 (44.3)	116 (29.2)
Microabrasion is too aggressive for WSCL treatment **	41 (10.3)	130 (32.7)	100 (25.2)	98 (24.7)	28 (7.1)
Infiltration increases the probability of pigmented plaque formation **	11 (2.8)	46 (11.6)	125 (31.5)	153 (38.5)	62 (15.6)

SA—strongly agree; A—agree; N—neutral; D—disagree; SD—strongly disagree; WSCLs—white spot caries lesions; *—positively phrased statements (ranging from 5—“strongly agree” to 1—“strongly disagree”); **—reverse-scored negative statements (ranging from 1—“strongly agree” to 5—“strongly disagree”).

**Table 5:** Attitude scores regarding the management of WSCLs.

Sociodemographic Data	Attitude	
	Mean (sd)	Median (IQR)
Total	13.5 (2.6)	14 (12; 15)
Gender		
Male	13.1 (2.6)	12 (11; 14)
Female	13.5 (2.6)	14 (12; 15)
	Kruskal–Wallis chi-squared = 3.5563, df = 1, *p*-value = 0.05932	
Specialty		
Dental therapist	13.0 (2.7) ^a^	13 (12; 14)
Pediatric dentist	14.4 (2.6) ^b^	15 (12; 15)
Orthodontist	13.8 (2.1) ^b^	14 (12; 15)
General dentist	13.5 (2.4) ^a,b^	13 (12; 15)
Prosthodontist	11.5 (1.2) ^a^	11 (10.5; 12.5)
Dental surgeon	14.8 (0.9) ^b^	15 (14; 15.5)
Dental hygienist	13.8 (3.4) ^a,b^	13 (11; 17)
	Kruskal–Wallis chi-squared = 25.301, df = 6, *p*-value < 0.001	
Years of clinical experience		
<5	13.7 (2.7) ^a^	14 (12; 15)
5–10	14.0 (2.4) ^a^	14 (12; 16)
11–20	12.9 (2.6) ^b^	14 (12.25; 15)
>20	12.2 (2.0) ^a,b^	12 (10; 14)
	Kruskal–Wallis chi-squared = 19.628, df = 3, *p*-value < 0.001	
Negative	54 (13.6)	
Positive	343 (86.4)	

IQR—interquartile range; sd—standard deviation; df—degrees of freedom, ^a,b^—mean values within the same column followed by different superscript letters differ significantly; means sharing a common letter are not significantly different.

**Table 6:** Self-reported clinical practice patterns in the diagnosis and management of WSCLs.

Item	Variants	Answers, *n* (%)
How often do you diagnose patients with WSCLs?	Less than 10% of cases	96 (24.2)
	10–20% of cases	73 (18.4)
	20–30% of cases	91 (22.9)
	30–40% of cases	66 (16.6)
	More than 40% of cases	64 (16.1)
	Never	7 (1.8)
What methods of WSCL diagnosis do you use?	Visual examination	357 (89.9)
	Staining	227 (57.2)
	Fluorescent diagnosis	101 (25.4)
	Polarization microscopy	7 (1.8)
	Probing	236 (59.4)
What WSCL treatments do you use?	Infiltration	183 (46.1)
	Microabrasion	20 (5.0)
	Remineralization with CPP-ACP	268 (67.5)
	Remineralization with nano-hydroxyapatite	105 (26.4)
	Remineralization with fluoride	247 (62.2)
	Composite restoration	60 (15.1)
	Monitoring	206 (51.9)
	Refer to a relevant specialist	41 (10.3)
	Other	5 (1.3)
Which WSCL treatment do you prefer in an esthetically significant area?	Infiltration	139 (35.0)
	Microabrasion	21 (5.3)
	Remineralization with CPP-ACP	58 (14.6)
	Remineralization with nano-hydroxyapatite	12 (3.0)
	Remineralization with fluoride	41 (10.3)
	Composite restoration	61 (15.4)
	Monitoring	16 (4.0)
	Refer to a relevant specialist	44 (11.1)
	Other	5 (1.3)

WSCLs—white spot caries lesions; CPP-ACP—casein phosphopeptide–amorphous calcium phosphate.

## Data Availability

The datasets used and analyzed during the current study are available from the corresponding author upon reasonable request.
